# Quality of life impairment in adult Moyamoya patients—preoperative neuropsychological assessment and correlation to MRI and H_2_^15^O PET findings

**DOI:** 10.1007/s10143-021-01660-9

**Published:** 2021-10-20

**Authors:** Patrick Haas, Monika Fudali, Sophie S. Wang, Helene Hurth, Till-Karsten Hauser, Ulrike Ernemann, Marcos Tatagiba, Nadia Khan, Constantin Roder

**Affiliations:** 1 Department of Neurosurgery and Moyamoya Center, University of Tübingen, Hoppe-Seyler-Straße 3, 72076 Tübingen, Germany; 2 Department of Neuroradiology, University of Tübingen, Hoppe-Seyler-Straße 3, 72076 Tübingen, Germany; 3 Moyamoya Center, University Children’s Hospital and University of Zürich, Steinwiesstrasse 75, 8032 Zürich, Switzerland

**Keywords:** Cerebral revascularization, Moyamoya disease, Positron emission tomography, Neuropsychology, Quality of life, Depression

## Abstract

Moyamoya angiopathy (MMA) related cerebral perfusion deficits or infarctions might influence quality of life (QoL). This study examines preoperative QoL in adult patients with MMA and correlates these with findings obtained via diagnostic imaging. Sixty-seven adult Moyamoya patients underwent preoperative neuropsychological testing including questionnaires to determine QoL, as well as psychiatric and depressive symptoms. The results were checked for correlation with territorial hypoperfusions seen in H_2_^15^O PET with acetazolamide (ACZ) challenge (cerebrovascular reserve) and infarction patterns observed in MRI. Each vascular territory was analyzed separately and correlated with QoL. Physical role function was restricted in 41.0% of cases and emotional role function in 34.4% of cases (SF-36). Obsessive–compulsive disorder (39.3%) (SCL-90-R), psychoticism (34.4%) (SCL-90-R), and depression (32.7%) (BDI-II) were also very common. Psychoticism was significantly more frequent in cases where perfusion deficits in PET CT were observed in both MCA territories (left *p* = 0.0124, right *p* = 0.0145) and infarctions in MRI were present in the right MCA territory (*p* = 0.0232). Depression was significantly associated with infarctions in the right MCA territory (SCL-90-R *p* = 0.0174, BDI-II *p* = 0.0246). Women were affected more frequently by depression (BDI-II, *p* = 0.0234). Physical role function impairment was significantly associated with perfusion deficits in the left MCA territory (*p* = 0.0178) and infarctions in the right MCA territory (*p* = 0.0428). MMA leads to impairments in different areas of QoL. Approximately one-third of all adult MMA patients suffered from depression, with women being most affected. In addition to depression, presence of executive dysfunctions and mental disorders such as psychoticism, obsessive–compulsive disorder, and impaired physical and emotional role function affected QoL. These patients showed significantly more often infarctions and perfusion deficits in the right MCA territory. Long-term studies with follow-up results are necessary to clarify a possible beneficial impact of early surgical revascularization on QoL and depression in adult MMA patients.

## Introduction

Moyamoya angiopathy (MMA) is a chronic and occasionally progressive steno-occlusive disease. It is primarily observed in the anterior circulation, but the posterior circulation may also be involved. It is characterized by a fine collateral network, akin to a puff of smoke in angiography [29]. MMA is more common in East Asian populations with a male-to-female ratio of approximately 1:1.8 to 2.2 and is characterized by two age peaks around 10 and 30–40 years [14]. Cerebral perfusion deficits resulting from the steno-occlusive changes lead to cerebrovascular events with ischemic or hemorrhagic stroke in the respective vascular territories.[27]. In addition to neurological deficits, neuropsychological deficits with cognitive impairment may be observed in MMA patients even in the absence of stroke [9, 12, 16, 20]. Depression, anxiety, and decreased quality of life (QoL) have previously been reported. [18, 25]. While the prevalence of mental disorders such as depression and QoL has been well studied for stroke survivors in general [4, 15], little information is available on the prevalence of neuropsychological disorders in MMA and their relation to stroke burden in MRI and perfusion deficits in PET.

Our study aims to investigate the neuropsychological status of MMA patients along with their QoL. Correlations to different types of perfusion deficits and infarction patterns are analyzed.

## Methods

A retrospective analysis of MMA patients with adult onset of the disease treated at our Moyamoya Center during 2014–2020 was performed. Inclusion criteria were confirmation of surgically untreated MMA by digital subtraction angiography (DSA), availability of MRI, age > 18, and preoperative completion of a neuropsychological assessment. As part of the routine preoperative evaluation, all patients received a standardized neuropsychological examination with testing of executive functions and QoL questionnaires under the supervision of a certified neuropsychologist. For preoperative imaging diagnostics, patients routinely received MRI and H_2_^15^0-PET with ACZ challenge. The visual analysis of the imaging data was blinded to the results of neuropsychological testing. Imaging and neuropsychological testing were performed within 3 months.

Ethical approval was obtained from the University of Tuebingen Ethics Committee. General patient data were obtained from the patients’ clinical files, any imaging data was stored and analyzed in the hospital’s PACS system.

### QoL and neuropsychological assessment

Four different questionnaires to determine quality of life (QoL), psychiatric symptoms and detect depressive symptoms were completed by patients during neuropsychological testing: The Short-Form 36 (SF-36) [30], the Symptom Checklist-90-R (SCL-90-R) [2], the Beck Depression Inventory (BDI-II) [26], and the Bern Bitterness Inventory (BVI) [33]. Results of the BDI-II were classified as mild depression at a score ≥ 14, moderate depression at ≥ 20, and major depression at ≥ 29. The items of the SF-36 included vitality, physical functioning, physical pain, general health perception, physical role function, emotional role function, social role function, and mental health. Results of the SF-36 were considered abnormal if the *z* score was less than − 1 SD. The BVI and the SCL-90-R were scored abnormal at a *t*-score greater than 60 (or greater than 63 for the Global Severity Index GSI of the SCL-90-R). The items of the SCL-90 included somatization, obsessive–compulsive disorder, interpersonal sensitivity, depression, anxiety, aggressiveness/hostility, paranoid ideation, phobic anxiety, psychoticism, and basic psychological distress.

Crystallized intelligence (IQ) was measured with the Multiple Choice Vocabulary Intelligence Test in German language (MWT-B), which is little affected by mild to moderate mental disorders and is therefore well suited for estimating premorbid intelligence levels such as those associated with mood disorders or depression [17].

In addition, the standard neuropsychological test battery of our Moyamoya Center includes 4 executive function tests: the Trail Making Test A (TMTA; psychomotor processing speed), the Trail Making Test B (TMTB; mental flexibility), the Chapuis Maze Test (Ch-L; problem solving), and the D2 Test (D2; selective attention). A dysexecutive cognitive syndrome (DCS) was defined as 2 or more abnormal test results, as described previously [23]. Handedness (right- and left-handed, ambidextrous) was assessed using the Edinburgh Handedness Inventory.

### *Imaging: MRI and H*_*2*_^*15*^*O PET with ACZ challenge*

H_2_^15^O PET was used to quantify cerebral perfusion reserve (CVR) in the three vascular territories, i.e., anterior cerebral artery (ACA), middle cerebral artery (MCA), and posterior cerebral artery (PCA) for both hemispheres, respectively. In addition to a baseline measurement, an acetazolamide (ACZ) challenge was used to identify perfusion deficits as described previously [8]. These deficits were classified into moderate, severe, and decrease/steal subtypes.

Magnetic resonance imaging Fluid Attenuated Inversion Recovery (FLAIR) and T2 sequences were applied to detect infarctions. These were again assigned to their vascular territories and divided into subtypes of less than 3 infarcts, equal to or greater than 3 infarcts with incipient confluence and large-scale infarctions. Additionally, we separated patients into two groups [23]: (1) a chronic disease group where the MRI showed signs of old ischemic lesions with the presence of larger gliotic changes and resulting brain atrophy and (2) a non-chronic disease group if the patients’ brain was without signs of chronic hypoperfusion/recurrent infarctions or atrophy in MRI (FLAIR).

### Data and statistical analysis

Study data was collected and managed using REDCap electronic data capture tools hosted by the University of Tuebingen [7]. Descriptive and mathematical statistics were performed using JMP (SAS Institute Inc. 2019, version 15.2.0, Cary, USA) and Excel (Microsoft Corporation, Version 2019, Redmond, USA). For nominal data, the chi-squared test or in cases of small sample sizes the 2-tailed Fisher-Yates exact test was applied. Effect size of significant results was determined by the associated phi coefficient: Φ < 0.30 was considered a small effect, Φ < 0.50 a moderate effect, and Φ ≥ 0.5 a strong effect. In the case of continuous values with a parametric distribution, the unpaired two-sample *t*-test was applied. Statistical significance was defined as *p* < 0.05. Normal distribution was checked by Q-Q plots and Shapiro–Wilk’s test.

## Results

Sixty-seven patients met the inclusion criteria. The median age was 38 years (interquartile range (IQR) 29.0) with a male-to-female ratio of 1:2.9 (17 men, 50 women). Fifty-three (79.1%) patients were right-handed, 12 (17.9%) ambidextrous, and 2 (3.0%) left-handed. Sixty-one (91.0%) patients were of Caucasian, 6 (9.0%) patients of Asian descent. Forty-one patients (61.2%) were classified as non-chronic and 26 (38.8%) as chronic. DCS was diagnosed in 28 (41.8%) patients. The response rate for the SCL-90-R and SF-36 was 61 (91.0%), for the BVI 58 (86.6%), and for the BDI-II 55 (82.1%) patients. IQ was determined in 44 (65.7%) patients (Table 1).

**Table 1:** Overview of the study cohort

Median age	38 (29)	
Handedness	67 tested patients	
- Right	- 53 (79.1%)	
- Ambidexter	- 12 (17.9%)	
- Left	- 2 (3.0%)	
Sex	67 patients	
- Male	- 17 (25.4%)	
- Female	- 50 (74.6%)	
Ethnicity	67 patients	
- Caucasian	- 61 (91.0%)	
- Asian	- 6 (9.0%)	
Disease status (MRI)	67 MRI datasets	
- Non-chronic	- 41 (61.2%)	
- Chronic	- 26 (38.8%)	
DCS	67 tested patients	
- Yes	- 28 (41.8%)	
- No	- 39 (58.2%)	
IQ	44 tested patients	
- < 85	- 3 (6.8)	
PET: perfusion deficit ACZ	62 PET datasets	
- Unilateral left	- 14 (22.6%)	
- Unilateral right	- 17 (27.4%)	
- Bilateral	- 31 (50.0.)	
- ACA left/right	- 26 (41.9%) / 28 (45.2%)	
- MCA left/right	- 42 (67.7%) / 47 (75.8%)	
- PCA left/right	- 4 (6.5%) / 4 (6.5)	
MRI: infarction	67 MRI datasets	
- Unilateral left	- 10 (14.9%)	
- Unilateral right	- 11 (16.4%)	
- Bilateral	- 33 (56.7%)	
- No infarctions	- 8 (11.9%)	
Infarction type	59 patients with infarctions	
- Hemorrhagic	- 3 (5.1%)	
- Ischamic	- 56 (94.9%)	
Infarction size	*ACA/MCA/PCA*	
	*Left*	*Right*
- < 3 lesions	- 12/16/2	- 8/16/1
- ≥ 3 lesions	- 13/21/0	- 13/21/1
- Confluent lesion	- 1/7/0	- 2/8/1

### Imaging data

A total of 402 vascular territories were analyzed in MR imaging and 372 in PET imaging, respectively. The most frequent infarctions were seen in the MCA territory (right 67.2%, left 65.7%), followed by the ACA (right 34.3%, left 38.8%) and PCA territory (right 4.5%, left 3.0%). There was no significant difference in laterality of infarctions: 10 (14.9%) patients were affected exclusively on the left and 11 (16.4%) on the right side, whereas 38 (56.7%) patients exhibited infarctions bilaterally.

**Fig. 1:**
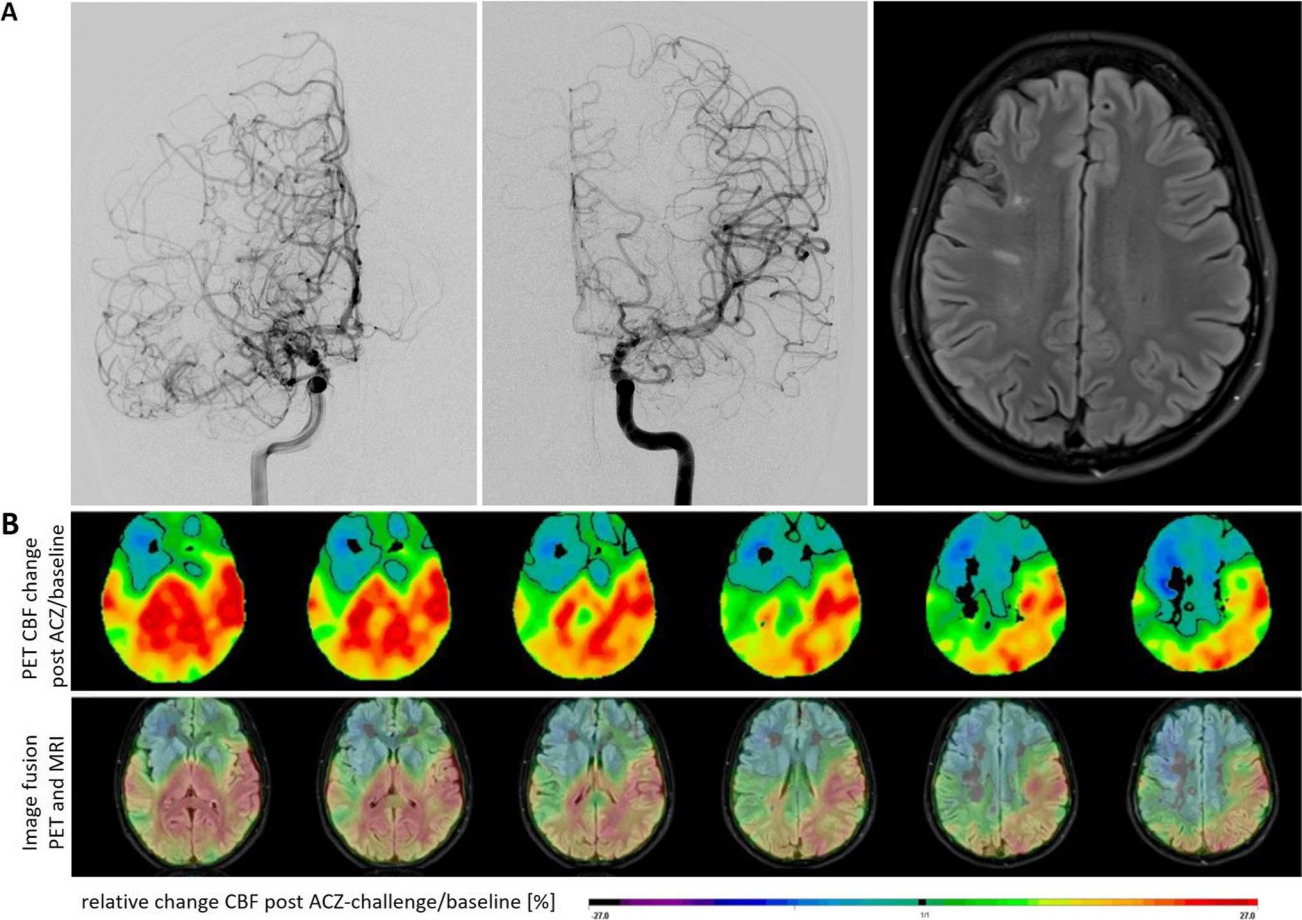
**A** upper row: Exemplary case of a 22-year-old female, right-handed, Caucasian patient with MMA at initial presentation after first manifestation with word-finding difficulties and short-term memory impairment. Neuropsychological testing revealed DCS with 3 of 4 abnormal test results. The BDI-II yielded 42 points, consistent with severe depression. The patient showed abnormal values in all subscales of the SCL-90-R and SF-36. Left: selective visualization of right ACI on angiography showing typical “puff of smoke” of as a correlate of a fine basal collateral network caused by occlusion of the MCA and stenosis of the A1 segment. Middle: Angiography of left ACI with hypoplastic A1 segment. Right: MRI T2 FLAIR sequence showing only small older infarctions in the right MCA territory. **B** lower row: Preoperative PET imaging with anatomical maps showing the perfusion reserve (in %) measured as difference between baseline measurement and after ACZ administration in H_2_^15^O PET

Five of 67 patients did not receive a H_2_^15^O PET examination. From 62 MMA patients examined by PET, 24 (38.7%) patients were without perfusion deficit in the baseline measurement. After ACZ challenge, a perfusion deficit not identified within baseline measurement was detected in 43 (69.4%) patients. Severe perfusion deficits occurred mainly in the right (*n* = 26) and left (*n* = 28) MCA territories, whereas a steal phenomenon occurred less often (*n* = 9 in ACA left or right and *n* = 9 in MCA left or right). Laterality of affected hemispheres did not vary significantly: 14 (22.6%) cases were unilaterally left, 17 (27.4%) unilaterally right, and 31 (50.0%) patients were affected bilaterally by perfusion deficits. An exemplary case can be seen in Fig. 1.

### QoL impairment and depression in relation to perfusion deficits in PET and infarctions seen in MRI

**Fig. 2:**
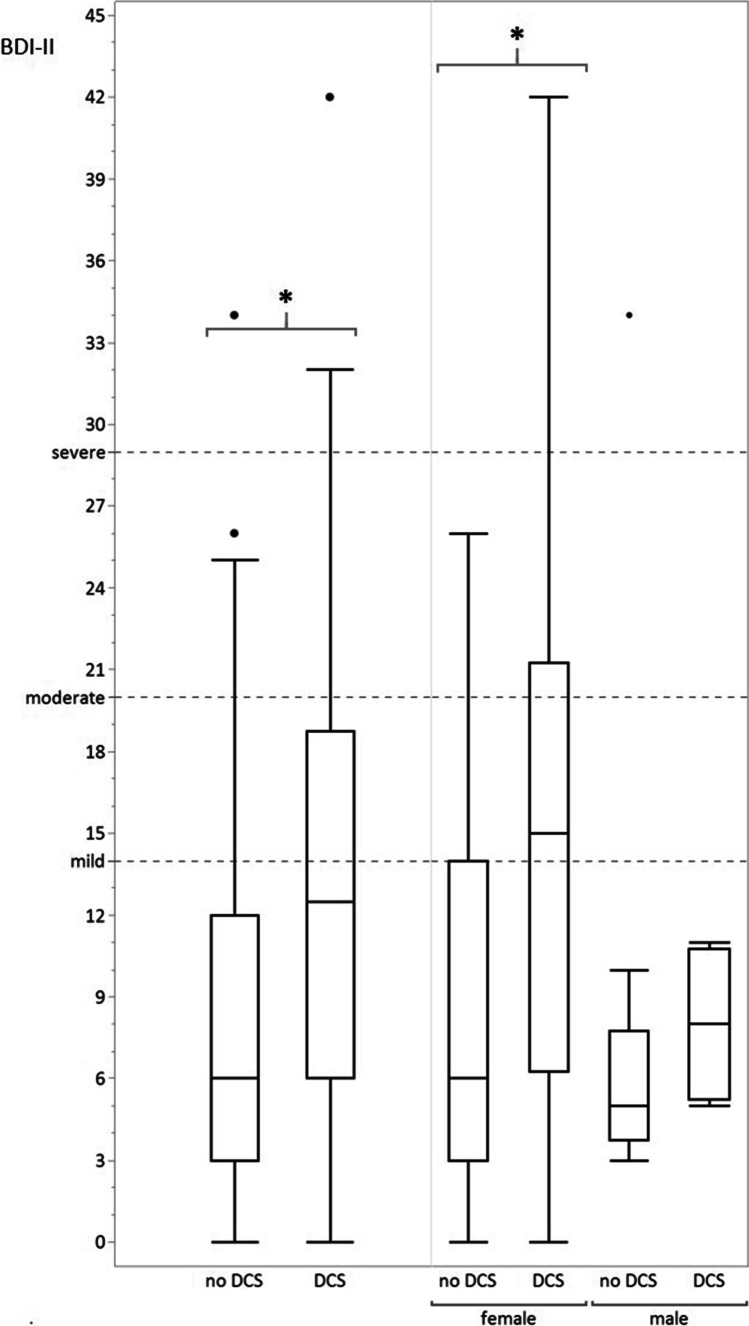
Box plots for Beck Depression Inventory (BDI-II) scores in female MMA patients with and without DCS. Female patients with DCS were significantly (*p* < 0.0315) affected by higher depression scores. Depression was classified as mild at ≥ 14, moderate at ≥ 20, and severe at ≥ 29 points

Fifty-five patients were screened using the BDI-II, 58 using the BVI and 61 using both the SCL-90-R and SF-36. The SCL-90-R detected depression in 18 (29.5%) patients, the BDI-II correspondingly in 17 (30.9%) patients (8 (14.5%) mild, 6 (10.9%) moderate, 3 (5.5%) severe). The sex-neutral parallel test reliability was significant (*p* < 0.0001). Overall, women were significantly more often affected by depression in the BDI-II (*p* = 0.0234, Φ = 0.30), especially if DCS was present (Fig. 2). However, this sex difference was not significant for the depression parameters in the SCL-90-R, since more men were classified as depressed by this test (BDI-II 16 women and 1 man out of 55 patients; SCL-90-R 14 woman and 4 men out of 61 patients). The presence of depression in the BDI-II as well as in the SCL-90-R was significantly associated with infarctions in the right MCA territory (*p* = 0.0246, Φ = 0.30 and *p* = 0.0174, Φ = 0.30, respectively). Also, the combined result of having a depression (SCL-90-R and BDI-II) was significantly more frequent in patients with infarctions in the right MCA territory (*p* = 0.0278, Φ = 0.28). A statistical correlation with infarction size (less than 3 infarcts, equal to or greater than 3 infarcts with incipient confluence, and large-scale infarctions) could not be found.

**Fig. 3:**
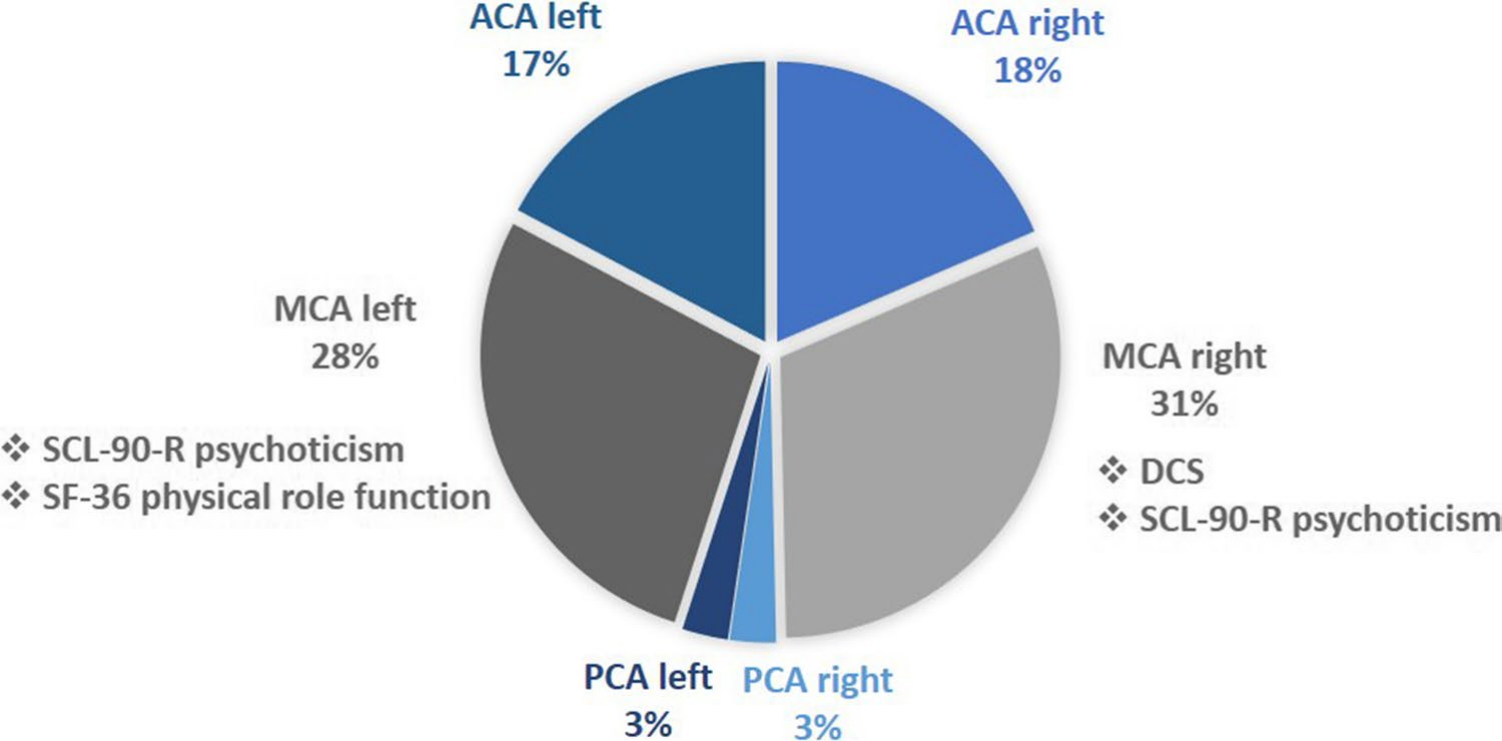
Pie chart showing percent frequency of perfusion deficits in PET/CT by vascular territory. Significant associated neuropsychological impairments (❖)

Furthermore, the prevalence for impaired physical role function (41.0%), obsessive–compulsive disorder (39.3%), psychoticism (34.4%) as well as abnormal emotional role function (34.4%), impaired general sense of health (36.0%), and vitality (32.8%) was high. Psychoticism was significantly more frequent in MMA patients with a perfusion deficit in the MCA territory (left *p* = 0.0124, Φ = 0.33; right *p* = 0.0145, Φ = 0.32) (Fig. 3) or infarctions in the right MCA territory (*p* = 0.0232, Φ = 0.29). The extent of the perfusion deficit (moderate, severe, decrease/steal) was statistically significant for both MCA territories (right *p* = 0.0155, Φ = 0.41; left *p* = 0.0066, Φ = 0.43). However, infarction size had no significant effect. The right MCA territory exhibited significantly more infarctions in MMA patients with reduced mental health (*p* = 0.0184, Φ = 0.30). Physical role function was significantly impaired for all affected patients with perfusion deficits present in the left MCA territory (*p* = 0.0178, Φ = 0.31) (Fig. 3) and infarctions in the right MCA territory (*p* = 0.0428, Φ = 0.25). There was no statistical correlation with infarction size or extent of perfusion deficit in either case. The Global Severity Index (GSI) of the SCL-90-R was pathologically elevated in 12 (19.7%) patients, but there was neither a significant association with specific perfusion deficits or infarction distribution, nor with the occurrence of DCS.

Neither the presence of infarctions as “yes or no” nor the subdivision non-chronic or chronic disease was significantly associated with a reduced QoL or the occurrence of depression. An abnormal GSI was not significantly associated with a specific perfusion deficit or infarct distribution or with the presence of DCS.

### Correlation of QoL and depression with DCS and IQ impairment

A DCS was seen in 28 (41.8%) of the 67 patients tested. Patients with chronic disease were significantly more likely to suffer from DCS than patients with a non-chronic manifestation according to MRI (*p* = 0.0020, Φ = 0.38). DCS occurred significantly more often if a perfusion deficit was only detected after ACZ challenge (*p* = 0.0442, Φ = 0.25) or in cases where infarctions in MRI were present in the right MCA territory (*p* = 0.0009, Φ = 0.40). Thereby a statistical correlation with the extent of perfusion deficit in the right MCA territory could be shown (*p* = 0.0003, Φ = 0.51). MMA patients with DCS suffered significantly more often from obsessive–compulsive disorder (*p* = 0.0181, Φ = 0.30), depression (*p* = 0.0404, Φ = 0.26), and psychoticism (*p* = 0.0030, Φ = 0.39). Furthermore, physical (*p* = 0.0072, Φ = 0.35) and emotional (*p* = 0.0142, Φ = 0.32) role function, as well as physical (*p* = 0.0072, Φ = 0.35) and social (*p* = 0.0236, Φ = 0.29) role functioning, was compromised in these patients. A possibly connected performance-related embitterment in the BVI was evident in 11% of all patients but did not reach statistical significance. The IQ of the 44 patients tested was distributed normally with a mean of 100.5 ± 12.0. Only 3 (6.8%) patients performed noticeably worse with IQ of < 85. These 3 patients all suffered from DCS. Patients with DCS had a significant lower IQ than patients without DCS (*p* = 0.0150). In contrast, the IQ between chronic and non-chronic disease did not vary significantly.

## Discussion

Measuring QoL in patients with MMA remains a challenge, mainly because the influence of individual extrinsic factors cannot be completely excluded. Neuropsychological impairments in correlation with perfusion deficits and infarctions are of particular interest in this context, as revascularization with extracranial-intracranial bypasses is an established technique that could potentially help to prevent further cognitive deterioration [13].

To date, only few studies have focused on the QoL of patients with MMA [25, 31]. To the authors’ knowledge, the present data represents the largest systematically collected cohort studied in this regard. The reported patient population is consistent with previously published data in terms of age structure and male-to-female ratio [5]. The perfusion deficits and infarctions were homogeneous and well balanced with respect to hemisphere allocation. The assignment of dominant vs. nondominant hemisphere played a minor role because surprisingly in the standardized determinations of handedness a significantly lower number of left-handers (2.0%) was found than would be expected in comparison with other populations (5–25.9%) [21]. A possible explanation would be a certain selection bias due to the higher number of female patients in our cohort, as their proportion of left-handers might be lower than that of men, as well as the already described larger geographic variations in prevalence [21].

An important quintessence of the available data is the normal distribution of IQ in adult patients with MMA and especially its independence from non-chronic or chronic disease status as seen in MRI. Festa et al. support these findings, although it should also be noted that this may not be applicable to pediatric manifestations of MMA [3]. Further results support this hypothesis, as Kronenburg et al. found in their meta-analysis a median IQ of 95 [min 94, max 99] [16]. This is an important finding for all affected adult MMA patients without DCS and should be used to reduce anxiety in patient education meetings. It should be noted that a lowered IQ is significantly related to a DCS. Whether one is the consequence of the other or vice versa might only be shown within long-term follow-up.

The data presented suggests that mental disorders such as anxiety and depression are more common in MMA patients, especially in women. This should be taken into account as part of a multimodal therapy concept. Liu et al. reported a prevalence of depression of 46.7% in their collective of 57 patients, Festa et al. of 28% in 29 patients examined, whereas Karzmark et al. found depression in 27.6% of 29 adult MMA patients without MRI evidence of stroke [3, 12, 18]. Our results yielded an intermediate value of 32.7% and 29.5%, respectively, so that depression might be assumed in about every third MMA patient and considering the significant male-to-female ratio especially in females. A possible above-average comorbidity of depression in MMA patients even without larger infarctions in MRI may have been underestimated in the past [12]. Yet, this comorbidity might become more relevant if compared to the estimated prevalence of 33% in patients after stroke (post-stroke depression (PSD)) [6]. The pathophysiology of PSD is poorly understood, but seems to be multifactorial: besides inflammatory processes, genetic and epigenetic aspects, cerebrovascular dysregulation, altered neuroplasticity, and alteration of neurotransmitters play a role [22]. Further studies are needed at this point to clarify which of these pathophysiological aspects play a key role in depression in MMA and how they are affected by the further course of the disease.

Decreased QoL in MMA patients has been investigated only by a few studies to date. Savolainen et al. concluded in their study of a Finnish cohort that QoL was decreased in both the physical and psychological domains of the WHOQOL score in 48 MMA patients compared with healthy controls [25]. Although there are methodological differences in the recording of QoL, our results seem to confirm these findings. In particular, the impaired physical and emotional role function, reduced vitality, and general sense of health seem to have a major impact. The elevated psychoticism scores in approximately one in three MMA patients in our study cohort were surprising, as clinical experience did not suggest such a finding. Together with the comparably high scores for impaired physical role function and compulsivity, these were significantly associated with DCS. Again, this does not establish causality and does not clarify the question whether a DCS develops first and results in the aforementioned mental disorders or whether both develop independently. Pending results of long-term follow-up observations of MMA patients are required for a better understanding.

Although the definition of a DCS remains inconsistent [3, 12, 16, 19], its occurrence as a sign of impaired executive performance is described as being the strongest associated (37%) impaired cognitive parameter in MMA patients by various authors. With a DCS in 41.8% of all cases, our present data is consistent with previously published reports [23]. Whether DCS is a consequence of chronic hypoperfusion or stroke or seems to appear independently remains unclear. Follow-up data of 26 non-surgically treated patients who were initially inconspicuous in terms of cognition showed an onset of mild cognitive impairment in 92% of cases after 1 year of follow-up and 100% affected cases after 2 years [28]. Our results suggest an association with the right hemisphere supplied by the MCA. While this association had already been observed in a much smaller cohort, a causality cannot be established yet [23]. A possible explanation for the debilitated nondominant hemisphere could be the impaired control of oculomotor function or hypoperfusion at the level of the basal ganglia, which is supported by the contribution of TMTB as a test of mental flexibility to the diagnosis of DCS [11]. Interestingly, in our analysis, the chronic MMA subgroup was more frequently diagnosed with DCS. Depression is a complex and multifactorial mental disorder that includes social as well as biological factors. Our results suggest an association with the occurrence of depression in adult MMA patients with a compromised right nondominant hemisphere in the MCA territory. However, it must be emphasized that a systematic review could not find any influence of lesion site or side, at least for PSD [1]. Whether results from PSD are applicable to MMA, however, remains unresolved to date. Possibly, the presented results are also an expression of the functional asymmetry and imbalance between a hyperactive right and a hypoactive left hemisphere proclaimed by some authors [10, 24].

### Limitations

Limitations of the study design must be mentioned. First, the data collection was retrospective, yet a very high data density could be achieved due to our routine testing of all Moyamoya patients. Furthermore, only preoperative data were evaluated, which does not allow to describe possible neurocognitive change after surgical revascularization. The different extent of infarctions as seen in some patients might be a factor influencing the evaluation of functional perfusion imaging (PET/CT). Zeifert et al. were able to show that revascularizing surgery does not lead to a significant deterioration of neurocognitive performance in a cohort of 85 patients with MMA [32]. Moreover, this study exclusively investigated adult and mainly Caucasian patients. The results are therefore not necessarily applicable to pediatric MMA patients and likewise only to a limited extent to other ethnicities.

H_2_^15^O PET was evaluated as a noninvasive method semi-quantitatively [8]. A differentiation between gray and white matter or cortical and subcortical lesion was not performed for the evaluation of MRI.

The neuropsychological test results can only be seen as a screening tool. A final diagnosis, such as depression, must always be made by a certified psychiatrist. However, all data collected for this study was evaluated by a certified neuropsychologist and might therefore be meaningful.

As described above, the definition of a DCS is inconsistent in the literature using different test batteries. Although individual subtests are identical, comparability among them is only possible to a limited extent.

In future studies, the correlation between impaired blood flow and brain anatomy (especially concerning brain lobes) must be further evaluated. Previous studies mainly focused on cerebral function and dysfunction in correlation to the brain lobes, but not the vascular supply which does not strictly follow the lobar structures.

## Conclusion

In the present study, we evaluated the preoperative neuropsychological outcomes with assessment of QoL, depression, and the presence of DCS in 67 adult patients with MMA and determined possible associations with infarctions seen in MRI and perfusion deficits in H_2_^15^O PET. Approximately one-third of all MMA patients suffer from depression, with women being most affected. QoL is significantly impaired in MMA patients, with executive dysfunction and mental disorders such as psychoticism, obsessive–compulsive disorder, and impaired physical and emotional role function being the main contributors in addition to depression. Affected patients suffered more frequently from infarctions and perfusion deficits in the right MCA territory. Long-term studies with follow-up results will be necessary to clarify the impact of surgical treatment on QoL and depression in adult MMA patients and whether specific neuropsychological disorders should support the indication for surgical revascularization.

## Data Availability

The datasets generated during and/or analyzed during this study are available from the corresponding author on reasonable request.
